# Prenatal IgE as a Risk Factor for the Development of Childhood Neurodevelopmental Disorders

**DOI:** 10.3389/fped.2021.601092

**Published:** 2021-05-14

**Authors:** Jennifer K. Straughen, Alexandra R. Sitarik, Christine Cole Johnson, Ganesa Wegienka, Dennis R. Ownby, Tisa M. Johnson-Hooper, Ghassan Allo, Albert M. Levin, Andrea E. Cassidy-Bushrow

**Affiliations:** ^1^Department of Public Health Sciences, Henry Ford Hospital, Detroit, MI, United States; ^2^Division of Allergy and Clinical Immunology, Department of Pediatrics, Medical College of Georgia at Augusta University, Augusta, GA, United States; ^3^Department of Pediatrics, Henry Ford Hospital, Detroit, MI, United States; ^4^Center for Autism and Developmental Disabilities, Henry Ford Hospital, Detroit, MI, United States; ^5^Department of Pathology, Henry Ford Hospital, Detroit, MI, United States

**Keywords:** attention deficit hyperactivity disorder, immunoglobulin E, neurodeveloment, pregnancy, cohort study

## Abstract

**Background:** Few studies have examined if maternal allergic disease is associated with an offspring's neurodevelopment. We hypothesized that Th-2 biased maternal immune function assessed as total serum immunoglobulin (Ig) E is associated with attention deficit hyperactivity disorder (ADHD).

**Methods:** Data are from the Wayne County Health, Environment, Allergy, and Asthma Longitudinal Study (WHEALS), a racially and socioeconomically diverse birth cohort in metropolitan Detroit, Michigan. Maternal total IgE was measured prenatally and at 1-month postpartum. Child total IgE was assessed at birth, 6 months, and 2 years of age. ADHD diagnosis was based on the parental report at the 10–12-year study visits or medical chart abstraction. Total IgE was log_2_ transformed. Poisson regression models with robust error variance were used to calculate the risk ratios (RR). Inverse probability weighting was used to correct for potential bias due to a loss to follow-up and non-response.

**Results:** Of the 636 maternal-child pairs in the analysis, 513 children were neurotypical and 123 had ADHD. Maternal prenatal total IgE was significantly associated with ADHD even after adjustment for potential confounders (RR = 1.08, 95% CI 1.03–1.13). Maternal and child IgE measures were positively and significantly correlated, but child total IgE was not associated with ADHD at any time point.

**Conclusions:** Maternal prenatal IgE may influence neurodevelopment, but additional studies are needed to confirm and expand these findings.

## Introduction

Neurodevelopmental disorders, such as autism spectrum disorder (ASD) and attention deficit hyperactivity disorder (ADHD), have risen in prevalence over the last 15–20 years. Current estimates suggest that one in 59 children are affected with ASD and 9% of children are affected with ADHD (1–3). Interestingly, increases in the prevalence of atopic disease, such as allergy and asthma, have also occurred during the same time period (4–6). Several authors have noted these concurrent trends as well as the high comorbidity of ASD/ADHD and atopic disease (7–11). However, evidence suggesting an association between neurodevelopmental disorders and atopic disease is inconsistent and stems largely from retrospective or cross-sectional studies (7–9, 11, 12).

There are important interactions between the immune and neural systems throughout life beginning in the prenatal period (13, 14). A growing body of research suggests that both atopic disease and neurodevelopmental disorders such as ADHD have their origins, at least in part, *in utero* (15, 16). Thus, it is possible that atopic disease and ADHD, two seemingly diverse outcomes, are both associated with early-life exposures that alter the immune environment during prenatal life. In support of this possibility, maternal prenatal allergy and asthma has emerged as a risk factor for both ASD and other developmental disorders in the offspring (17). In one Australian study of 420 healthy term infants, children born to mothers with a history of allergic disease were more likely to have an allergic disease and suboptimal neurodevelopmental outcomes at 12–18 months of age (18). Total serum concentrations of immunoglobulin (Ig) E are strongly but imperfectly associated with the presence of allergic diseases (19, 20). Maternal IgE levels during pregnancy are associated with a risk of atopic disease in the offspring, perhaps as a surrogate marker of the relative balance of maternal Th-1 and Th-2 immunity (20–22). However, maternal immune function and maternal factors associated with offspring atopy (i.e., IgE) have not been thoroughly examined in relation to ADHD. Investigating the possible relationships between the levels of IgE during critical windows of offspring development and before the onset of ADHD symptoms may offer important insights into the etiologies of this disorder.

Consequently, we hypothesize that ADHD is associated with an early life immune function or sensitization that does not necessarily result in an overt atopic disease, as measured by maternal prenatal total IgE in the prenatal and perinatal periods. The association between offspring early life and childhood total IgE through age two and ADHD are also examined. These analyses were conducted in a large prospective and racially/socioeconomically diverse birth cohort study from the Detroit metropolitan area in southeastern Michigan.

## Materials and Methods

### Study Population

The Wayne County Health, Environment, Allergy, and Asthma Longitudinal Study (WHEALS) recruited pregnant women with due dates from September 2003 through December 2007, and who were seeing a practitioner at one of five clinics in the Henry Ford Health System to establish a birth cohort (23, 24). All women were in their second trimester or later, aged 21–49 years, and living in a predefined geographic area in Wayne and Oakland counties that included the city of Detroit as well as the suburban areas immediately surrounding the city. Mothers were interviewed in the clinic prenatally and postpartum, and interviewer-administered questionnaires were completed at child age one, six, 12, and 24 months. Children and their parent/guardian were invited to return for a clinic visit at child age of 2 years and again at child age of 10–12 years for assessment of child health. All participants provided a written, informed consent and study protocols were approved by the Henry Ford Health System Institutional Review Board; at the age 10–12 year visit, children provided a written, informed assent.

Of the 1,258 maternal-child pairs in the WHEALS cohort, 1,201 had a maternal total IgE measurement at either the prenatal visit or 1-month postpartum visit. Of those, 651 maternal-child pairs were followed through 10 years of age and had information on neurodevelopmental outcomes. Seven children with a sensory processing disorder, but not ADHD, and eight children with ASD, but not ADHD, were removed prior to the analysis, leaving a total of 636 maternal-child pairs in the final analytic sample.

### Total IgE Measurement

Blood was collected from mothers during the prenatal visit and at the 1-month postpartum home visit, while blood was collected from children at birth (umbilical cord blood), 6-months, 1-year, and 2-years. Total IgE (IU/mL) was measured in all blood samples with the Pharmacia UniCAP system (Phadia, Portage, MI) using the manufacturer's standard protocols. Further details on the total IgE measurement have been previously published (23).

### Definition of ADHD and Neurotypical Development

At the age 10–12-year visit, the caregiver (95% the mother) reported if the child had ever been diagnosed with ADHD, ASD, Asperger's syndrome, or a sensory processing disorder. A question about Asperger's syndrome was asked separately since children may have been diagnosed prior to the Diagnostic and Statistical Manual of Mental Disorders, Fifth Edition (DSM-5). Caregiver report of a “suspect” diagnosis was also classified as positive for the primary analysis.

A subset of 325 WHEALS children who had received care from the Henry Ford Health System providers had their medical records abstracted for additional health information, including ADHD diagnoses. Caregiver report of ADHD diagnosis was validated within this subset using the kappa (κ) statistic to evaluate agreement. Landis and Koch (25) criteria were used to evaluate strength of agreement. As previously reported, there was a high degree of agreement between caregiver-reported ADHD and the medical record (κ = 0.84, 95% CI 0.78–0.91) (26). Given that only a subset of children had a medical record review and moderate to high level of agreement between caregiver report and the medical record, the statistical analysis classified a child as having ADHD if their caregiver-reported ADHD or if it was reported in the medical record.

Children with ADHD were compared to children who were considered neurotypical (NT) because they did not have a caregiver-reported neurodevelopmental diagnosis. Other neurodevelopmental disorders were not evaluated as a separate outcome due to the small sample sizes. To evaluate the robustness of the results, two sensitivity analyses were performed in addition to the primary analysis of all ADHD cases relative to NT: (1) ADHD cases with a “suspect” parental diagnosis but no chart abstracted diagnosis were removed, and (2) ADHD cases with a comorbid ASD diagnosis (defined via parental report or chart abstraction) were removed.

### Covariates

Maternal self-reported race, date of birth, marital status, household income, address, education, insurance type, parity, alcohol use, pet keeping, allergic disease, smoking history, and exposure to environmental tobacco smoke (ETS) were collected at the prenatal interview. Maternal prenatal care records were abstracted to obtain prescription antibiotic and antifungal use (27). Height and weight were also abstracted and body mass index (BMI; kg/m^2^) at the first prenatal care visit (mean gestational age measure at 9.1 ± 4.9 weeks; 83% were taken during the first trimester) were calculated. Although the first measured BMI in pregnancy and self-reported prepregnancy BMI have been shown to be highly correlated in other studies (28), the first measured BMI during pregnancy represents both prepregnancy body size and early pregnancy-related weight gain. Delivery records for WHEALS women were abstracted to obtain delivery mode, birth weight, and gestational age at delivery.

### Statistical Analysis

In order to describe the differences in children included and excluded from the analyses and to describe differences by ADHD status, analysis of variance was used for continuous covariates while chi-square tests were used for categorical covariates (unless any cell sizes were <5, in which case, the Fisher's exact test was used). For descriptive purposes, total IgE measurements were first compared on the untransformed IU/mL scale, with between group differences evaluated using the Wilcoxon rank sum test. In order to have meaningful coefficients in regression models (described further in the Results), total IgE measurements were log2-transformed prior to model inclusion. The log2 transformation was also used to examine Pearson correlations between total IgE measurements in order to meet normality assumptions. Supplementary Figure 1 demonstrates that both log2 and natural log transformation of the IgE values effectively normalized them. Because a loss to follow-up and non-response can affect the internal validity of estimates, inverse probability weighting (IPW) was used to correct for this bias (29). Analytic sample inclusion was used as the outcome in a logistic regression model with the following covariates: maternal age at delivery, maternal race, insurance type, household income, marital status, maternal education, location of residence (urban vs. suburban), maternal prenatal smoking, prenatal ETS, prenatal alcohol use, prenatal indoor pets, maternal allergic disease (allergies and asthma), mode of delivery, parity, child sex, gestational age at birth, and birth weight. The predicted probability of inclusion for each subject was extracted from this model; weights were calculated as the inverse of the “treatment” received. In other words, if *p* = probability of inclusion, then IPW = 1/p for included children and IPW = 1/(1-p) for excluded children. Covariate balance was assessed using the standardized differences (difference in means or proportions divided by standard error) before and after weighting, with imbalance defined as absolute value >0.20.

To evaluate the association between maternal total IgE (prenatal and at 1 month postpartum) and ADHD in offspring, risk ratios (RR) were obtained from Poisson regression models using a robust error variance (30). In all the models, subjects were weighted using the IPW described previously. Models were evaluated both before and after adjusting for potential confounders. Potential confounders were selected as those that were significantly associated with maternal total IgE and ADHD and included maternal age at birth, household income, marital status, maternal education, maternal prenatal smoking, household prenatal smoke exposure, prenatal pet exposure, maternal prenatal BMI, prenatal antibiotic use, prenatal antifungal use, first born child, mode of delivery, gestational age at birth, birthweight z-score, child sex, child race, and breastfeeding status at 1-month. From this pre-specified set of hypothesized confounders, child sex and prenatal antifungal use met these criteria. Child sex and breastfeeding status at 1-month were *a priori* specified as potential effect modifiers and were tested using multiplicative interaction terms.

Because there was covariate missingness in the analytic dataset, multiple imputation was performed in addition to a complete-case analysis. A total of 20 imputed datasets were calculated using all exposure variables, the outcome of interest, confounders, and the IPW for loss to follow-up. The SAS procedure MI (SAS Institute Inc., Cary, NC) with the fully conditional specification algorithm was used to generate imputed datasets; imputation quality was assessed via trace plots and variance information (31). The SAS *mianalyze* procedure was used to pool estimates.

## Results

Among the 636 maternal-child pairs included in the analysis, 123 (19.1%) were diagnosed with ADHD and 513 (79.7%) were NT. Ten children had both ADHD and ASD. The comparison of children included and excluded from analyses are shown in Table 1. Children included in the analysis were more likely to have mothers with Health Alliance Plan coverage (an insurance company owned by the Henry Ford Health System), higher household incomes, at least a bachelor's degree, and were more likely to be married and live in a suburban residence (all *p* < 0.05). Included mothers were also older on average and less likely to smoke or be exposed to tobacco smoke during pregnancy; included children on average also weighed more at birth. Standardized differences of these associations were as large as 0.75; however, after weighting the subjects by their IPW for loss to follow-up, no significant associations were found, and the absolute value of all standardized differences were <0.20 (maximum = 0.04), which suggested that balance was achieved in these covariates between the included and excluded subjects after weighting.

**Table 1:** Description of maternal-child pairs included and excluded from the analysis.

**Covariate**	**Level**	**Excluded*N* = 622**	**Included*N* = 636**	**Before IPW**^**a**^		**After IPW**^**a**^	
		***N*** **(Column %) or** ***N*****, Mean** **±** **SD**		***p*-value^b^**	**D^c^**	***p*-value^b^**	**D^c^**
Race-ethnicity of mother	Caucasian	137 (22%)	153 (24.1%)	0.76	0.074	0.98	0.041
	African American	394 (63.3%)	384 (60.4%)				
	Hispanic	38 (6.1%)	40 (6.3%)				
	Arabic	30 (4.8%)	29 (4.6%)				
	Other/Mixed	23 (3.7%)	30 (4.7%)				
Insurance coverage	Health Alliance Plan	172 (27.7%)	330 (51.9%)	**<0.001**	0.751	0.99	0.001
	Other insurance	198 (31.8%)	233 (36.6%)				
	No insurance	9 (1.4%)	6 (0.9%)				
	Refused/do not know/missing	243 (39.1%)	67 (10.5%)				
Household income	<$20,000	116 (18.6%)	66 (10.4%)	**<0.001**	0.338	0.99	0.036
	$20,000–$40,000	147 (23.6%)	148 (23.3%)				
	$40,000–$80,000	175 (28.1%)	172 (27%)				
	$80,000–$100,000	49 (7.9%)	86 (13.5%)				
	≥$100,000	59 (9.5%)	89 (14%)				
	Refused to answer	76 (12.2%)	75 (11.8%)				
Mother married	No	270 (43.4%)	215 (33.8%)	**<0.001**	0.198	0.51	0.026
	Yes	352 (56.6%)	421 (66.2%)				
Maternal education	<High school diploma	51 (8.2%)	23 (3.6%)	**<0.001**	0.352	0.97	0.030
	High school diploma	132 (21.2%)	96 (15.1%)				
	Some college	312 (50.2%)	293 (46.1%)				
	≥Bachelor's degree	127 (20.4%)	224 (35.2%)				
Maternal age at birth (years)		622, 29.1 ± 5.2	636, 30.0 ± 5.2	**0.001**	0.185	0.90	0.005
Location of residence	Suburban	254 (40.8%)	301 (47.3%)	**0.020**	−0.131	0.84	−0.008
	Urban	368 (59.2%)	335 (52.7%)				
Mom smoked during pregnancy	No	526 (84.6%)	582 (91.5%)	**<0.001**	−0.215	0.52	0.026
	Yes	96 (15.4%)	54 (8.5%)				
Prenatal ETS exposure	No	426 (68.5%)	485 (76.3%)	**0.002**	−0.174	0.72	0.014
	Yes	196 (31.5%)	151 (23.7%)				
Prenatal alcohol use	No	596 (96.1%)	610 (96.1%)	0.95	0.004	0.81	−0.009
	Yes	24 (3.9%)	25 (3.9%)				
Prenatal indoor dogs	No	485 (78%)	470 (73.9%)	0.091	0.095	0.85	−0.008
	Yes	137 (22%)	166 (26.1%)				
Prenatal indoor cats	No	528 (84.9%)	527 (82.9%)	0.33	0.055	0.42	0.032
	Yes	94 (15.1%)	109 (17.1%)				
Maternal doctor diagnosed hay fever or allergic rhinitis	No	526 (85.3%)	528 (84.6%)	0.75	0.013	0.93	0.001
	Yes	91 (14.7%)	96 (15.4%)				
Maternal doctor diagnosed asthma	No	498 (80.1%)	507 (79.8%)	0.92	0.005	0.87	−0.007
	Yes	124 (19.9%)	128 (20.2%)				
Mode of delivery	Vaginal	386 (62.7%)	398 (62.8%)	0.97	0.003	0.74	0.015
	C-section	230 (37.3%)	236 (37.2%)				
First born child	No	413 (66.4%)	385 (60.5%)	**0.031**	0.122	0.96	0.002
	Yes	209 (33.6%)	251 (39.5%)				
Child sex	Male	305 (49.1%)	317 (49.8%)	0.80	−0.016	0.58	0.021
	Female	316 (50.9%)	319 (50.2%)				
Gestational age at delivery (weeks)		604, 38.7 ± 1.8	628, 38.7 ± 1.7	0.56	0.030	0.95	−0.004
Birth weight (grams)		576, 3,259 ± 556	606, 3,347 ± 587	**0.009**	0.148	0.91	−0.005

*ETS, environmental tobacco smoke; SD, standard deviation*.

a *Inverse probability weighting (IPW) to account for loss to follow-up*.

b *Calculated by analysis of variance for numerical covariates and chi-square test for categorical covariates*.

c *Standardized difference, defined as the difference in means or proportions divided by standard error*.

*The bold values indicate statistical significance*.

The association between maternal and child characteristics and ADHD was also examined (Table 2). Maternal prenatal BMI, maternal report of doctor diagnosed hay fever or allergic rhinitis, maternal doctor diagnosed asthma, prenatal antifungal use, child sex, and gestational age at delivery all differed significantly between children with ADHD relative to NT children (all *p* < 0.05). Specifically, relative to NT children, children with ADHD had mothers with higher prenatal BMIs, were more likely to use prenatal prescription antifungals and more likely to have a self-reported history of hay fever or allergic rhinitis and asthma. Children with ADHD were more likely to be male and on average had earlier gestational ages at delivery. Of these characteristics, only prenatal antifungal use and child sex were also significantly associated with maternal total IgE (Supplementary Table 1).

**Table 2:** Maternal and child characteristics associated with ADHD (*N* = 636).

**Covariate**	**Level**	**NT*N* = 513**	**ADHD^a^*N* = 123**	***p*-value**
		***N*** **(Column %) or** ***N*****, Mean** **±** **SD**		
Household income	<$20,000	50 (9.7%)	16 (13%)	0.21^b^
	$20,000–$40,000	113 (22%)	35 (28.5%)	
	$40,000–$80,000	138 (26.9%)	34 (27.6%)	
	$80,000–$100,000	72 (14%)	14 (11.4%)	
	≥$100,000	79 (15.4%)	10 (8.1%)	
	Refused to answer	61 (11.9%)	14 (11.4%)	
Mother married	No	169 (32.9%)	46 (37.4%)	0.35^b^
	Yes	344 (67.1%)	77 (62.6%)	
Maternal education	<High school diploma	20 (3.9%)	3 (2.4%)	0.067^c^
	High school diploma	76 (14.8%)	20 (16.3%)	
	Some college	225 (43.9%)	68 (55.3%)	
	≥Bachelor's degree	192 (37.4%)	32 (26%)	
Maternal age at birth (years)		513, 30.2 ± 5.2	123, 29.6 ± 5.3	0.26^d^
Maternal BMI-first measured in pregnancy		487, 30.2 ± 7.5	109, 32.5 ± 9.1	**0.004**^**d**^
Mom smoked during pregnancy	No	471 (91.8%)	111 (90.2%)	0.58^b^
	Yes	42 (8.2%)	12 (9.8%)	
Prenatal ETS exposure	No	397 (77.4%)	88 (71.5%)	0.17^b^
	Yes	116 (22.6%)	35 (28.5%)	
Prenatal indoor pets	No	327 (63.7%)	72 (58.5%)	0.28^b^
	Yes	186 (36.3%)	51 (41.5%)	
Prenatal antibiotic use	No	204 (46.6%)	46 (44.2%)	0.67^b^
	Yes	234 (53.4%)	58 (55.8%)	
Prenatal antifungal use	No	366 (83.6%)	72 (69.2%)	**<0.001**^**b**^
	Yes	72 (16.4%)	32 (30.8%)	
Child sex	Male	227 (44.2%)	90 (73.2%)	**<0.001**^**b**^
	Female	286 (55.8%)	33 (26.8%)	
Race-ethnicity of child	White	110 (21.4%)	28 (22.8%)	0.40^b^
	African American	310 (60.4%)	79 (64.2%)	
	Other/Mixed	93 (18.1%)	16 (13%)	
First born child	No	319 (62.2%)	66 (53.7%)	0.082^b^
	Yes	194 (37.8%)	57 (46.3%)	
Mode of delivery	Vaginal	324 (63.3%)	74 (60.7%)	0.59^b^
	C-section	188 (36.7%)	48 (39.3%)	
Gestational age at delivery (weeks)		508, 38.8 ± 1.6	120, 38.3 ± 2.0	**0.003**^**d**^
Birthweight *z*-score		490, −0.04 ± 1.0	111, −0.04 ± 0.99	0.96^d^
Breastfeeding status at 1-month	Not breastfed	101 (20.3%)	24 (20.5%)	0.95^b^
	Mixed feeding	323 (64.9%)	77 (65.8%)	
	Breastfeeding only	74 (14.9%)	16 (13.7%)	
Maternal doctor diagnosed hay fever or allergic rhinitis	No	438 (86.2%)	90 (77.6%)	**0.020**^**b**^
	Yes	70 (13.8%)	26 (22.4%)	
Maternal doctor diagnosed asthma	No	418 (81.6%)	89 (72.4%)	**0.021**^**b**^
	Yes	94 (18.4%)	34 (27.6%)	
Mother prenatal total IgE (IU/mL)		385, 33.7 ± 4.1^e^	99, 49.2 ± 4.4^e^	**0.005**^**f**^
Mother 1-month total IgE (IU/mL)		489, 36.7 ± 4.4^e^	116, 52.9 ± 4.9^e^	**0.005**^**f**^

*ADHD, attention deficit hyperactivity disorder; BMI, body mass index; ETS, environmental tobacco smoke; Ig, immunoglobulin; NT, neurotypical; SD, standard deviation*.

a *includes 10 children with both ADHD and ASD*.

b *Calculated by the chi-square test*.

c *Calculated by Fisher's exact test (at least one cell size <5)*.

d *Calculated by analysis of variance*.

e *Geometric mean and standard deviation*.

f *Calculated by the Wilcoxon rank sum test*.

*The bold values indicate statistical significance*.

Table 3 presents the associations between maternal total IgE and ADHD. In the primary analysis including all 123 ADHD cases, both measures of maternal total IgE were significantly associated with ADHD prior to covariate adjustment (Table 3). Specifically, for each 2-fold increase in the maternal prenatal total IgE, the risk of ADHD increased by 10% (Model 1; RR 1.10, 95% CI 1.05–1.15; *p* < 0.001); similarly, for each 2-fold increase in the maternal 1-month total IgE, the risk of ADHD increased by 8% (Model 1; RR 1.08, 95% CI 1.04–1.13; *p* < 0.001). After adjusting for covariates in a complete-case analysis, both maternal prenatal (Model 2; RR 1.07, 95% CI 1.01–1.12; *p* = 0.014) and 1-month (Model 2; RR 1.06, 95% CI 1.01–1.11; *p* = 0.025) IgE remained statistically significantly associated with ADHD. However, only maternal prenatal IgE remained statistically significant in the multiple imputation estimates (Model 3; RR 1.08, 95% CI 1.03–1.13; *p* = 0.001). In the first sensitivity analysis removing seven children who had a “suspect” ADHD diagnosis, results for the maternal prenatal IgE were effectively unchanged (Model 3; RR 1.09, 95% CI 1.04–1.14, *p* < 0.001). Results were again consistent in the second sensitivity analysis where we repeated the analysis after removing 10 children who had both ASD and ADHD. Specifically, effect estimates with prenatal IgE were not impacted and again remained statistically significantly associated with ADHD (Model 3; RR 1.08, 95% CI 1.03–1.13, *p* < 0.001). In both sensitivity analyses, there remained no association between the 1-month postpartum IgE and ADHD. Additionally, child sex and breastfeeding status at 1-month did not significantly modify any of the associations between maternal IgE and ADHD (all interaction *p* ≥ 0.20).

**Table 3:** Association between the maternal total IgE measurements and ADHD in the offspring.

**Exposure**		**Model 1**^**a**^			**Model 2**^**b**^			**Model 3**^**c**^	
	**N**	**RR (95% CI)^d^**	***p*-value**	***N***	**RR (95% CI)^d^**	***p*-value**	***N***	**RR (95% CI)^d^**	***p*-value**
**Full analysis**^**e**^									
Mother prenatal total IgE	484	1.10 (1.05, 1.15)	**<0.001**	404	1.07 (1.01, 1.12)	**0.014**	636	1.08 (1.03, 1.13)	**0.001**
Mother 1-month total IgE	605	1.08 (1.04, 1.13)	**<0.001**	520	1.06 (1.01, 1.11)	**0.025**	636	1.03 (0.96, 1.10)	0.37
**Sensitivity analysis #1**^**f**^									
Mother prenatal total IgE	479	1.11 (1.06, 1.17)	**<0.001**	400	1.08 (1.02, 1.14)	**0.007**	629	1.09 (1.04, 1.14)	**<0.001**
Mother 1-month total IgE	599	1.10 (1.05, 1.15)	**<0.001**	515	1.07 (1.01, 1.12)	**0.011**	629	1.04 (0.97, 1.13)	0.30
**Sensitivity analysis #2**^**g**^									
Mother prenatal total IgE	479	1.10 (1.05, 1.15)	**<0.001**	400	1.08 (1.02, 1.14)	**0.007**	626	1.08 (1.03, 1.13)	**<0.001**
Mother 1-month total IgE	595	1.09 (1.04, 1.14)	**<0.001**	511	1.07 (1.02, 1.12)	**0.009**	626	1.03 (0.96, 1.11)	0.36

*ADHD, attention deficit hyperactivity disorder; Ig, immunoglobulin; NT, neurotypical; RR, risk ratio*.

a *Inverse probability weights; unadjusted; complete-case analysis*.

b *Inverse probability weights; adjusted for child sex and prenatal antifungal use; complete-case analysis*.

c *Inverse probability weights; adjusted for child sex and prenatal antifungal use; multiple imputation analysis*.

d *Maternal total IgE measurements log2-transformed prior to analysis so that risk ratios are interpreted as the increase in risk of ADHD associated with a 2-fold increase in maternal total IgE*.

e *All ADHD cases (N = 123) vs. NT (N = 513)*.

f *ADHD cases with a “suspect” parental diagnosis have been removed (N = 7), leaving N = 116 ADHD cases vs. N = 513 NT for analysis*.

g *ADHD cases with a comorbid ASD diagnosis have been removed (N = 10), leaving N = 113 ADHD cases vs. N = 513 NT for analysis*.

*The bold values indicate statistical significance*.

**Figure 1:**
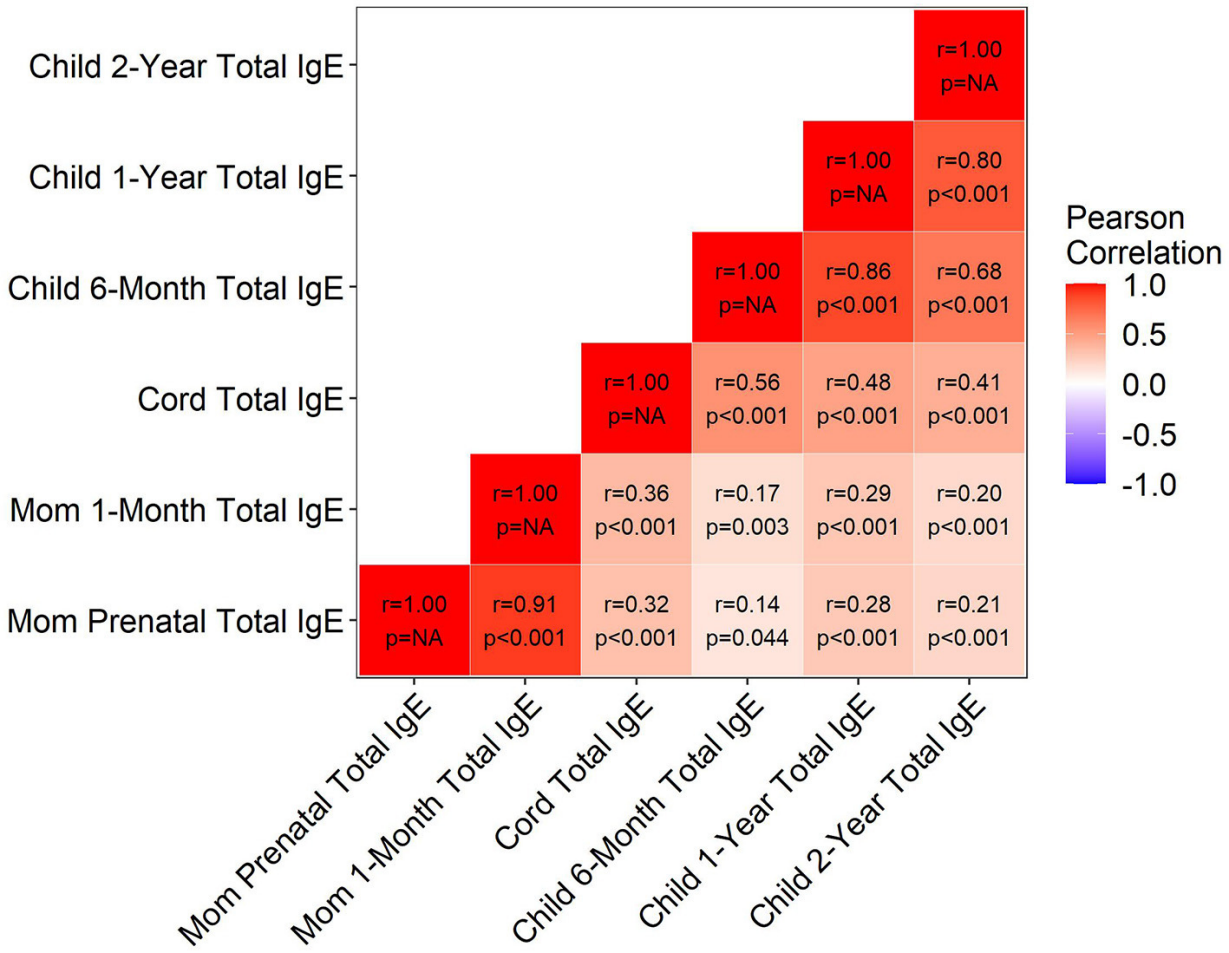
Correlation between the maternal and child total IgE measurements. Ig, immunoglobulin.

The correlations between maternal and child total IgE measurements are shown in Figure 1. As expected, measures were positively and significantly correlated, both within maternal prenatal and postnatal time points (*r* = 0.91), within the child at all time points (*r* minimum = 0.41, maximum = 0.86), and between mother and child at all time points (*r* minimum = 0.14, maximum = 0.36). Consistent with what we observed in Figure 1, all child total IgE measurements were significantly positively associated with the maternal prenatal total IgE, even after IPW and covariate adjustment (Table 4). However, regardless of age at IgE assessment, child total IgE was not significantly associated with ADHD in multivariable models (Table 4). For example, for each 2-fold increase in child 2-year total IgE, there was a 4% higher risk of ADHD, but this was not significant (*p* = 0.38).

**Table 4:** Associations between child total IgE and maternal prenatal total IgE and ADHD.

**Child Total IgE Measurement**	**Association with Mother Prenatal Total IgE**		**Association with ADHD (vs NT)**	
	**β (95% CI)^a^**	***p*-value**	**RR (95% CI)^b^**	***p*-value**
Cord	0.31 (0.22, 0.40)	**<0.001**	1.01 (0.94, 1.08)	0.85
6-months	0.14 (0.01, 0.28)	**0.047**	1.00 (0.88, 1.13)	0.98
1-year	0.26 (0.14, 0.39)	**<0.001**	1.01 (0.91, 1.13)	0.81
2-years	0.15 (0.04, 0.26)	**0.006**	1.04 (0.96, 1.12)	0.38

*ADHD, attention deficit hyperactivity disorder; Ig, immunoglobulin; NT, neurotypical; RR, risk ratio*.

a *Interpreted as the mean increase in log2-transformed child total IgE, for a 1-unit increase in log2-transformed maternal prenatal total IgE, after adjusting for child sex and prenatal antifungal use. Inverse probability weighting was used in all models*.

b *Change in outcome risk associated with a 2-fold increase in child total IgE, after adjusting for maternal prenatal total IgE, child sex, and prenatal antifungal use. Inverse probability weighting was used in all models*.

*The bold values indicate statistical significance*.

## Discussion

The findings from this study suggest that maternal IgE during pregnancy influences the risk of ADHD in an offspring. Specifically, higher levels of maternal prenatal total IgE were associated with a statistically significant increased risk of ADHD diagnosis in the offspring, which remained after covariate adjustment. Child IgE throughout the first 2 years of life was not associated with ADHD. These findings are significant because they highlight maternal prenatal total IgE level as an important potential marker of risk of ADHD. To our knowledge, few studies have examined prenatal exposure to immunoglobulins generally and risk of ADHD or other neurodevelopmental disorders such as ASD. While results of other studies are not directly comparable to the findings presented here, inferences may be drawn from similar work on other neurodevelopmental outcomes. Grether et al. studied maternal prenatal total IgG and total IgM and found no association with the risk of ASD (32). However, IgE is functionally distinct from IgG and IgM and results from different types of immune responses. Higher levels of IgE are associated with allergic disease and other responses (20, 33). As such, different associations between ADHD or ASD and immunoglobulin type are plausible.

Furthermore, previous studies suggested that maternal IgE is correlated with infant IgE (21, 34). In this analysis, we found similar correlations, yet, in our study the infant total IgE levels during early postnatal life were not associated with ADHD. Other studies that have examined IgE in childhood and neurodevelopment concurrently reported similar findings (35, 36). Verlaet et al. found no association between the total IgE and ADHD when IgE was measured in 6–12-year-old children with and without ADHD (35). On the other hand, Wang et al. found a positive association between the total IgE and ADHD measured cross-sectionally in children 8–10 years of age (12). The different age distributions in these studies may explain some of the conflicting findings. Future studies should examine the rate of change of IgE across infancy and childhood. Given the high comorbidity between atopic disease and neurodevelopmental diseases (7–11), it is somewhat surprising that in our data, there is no association between IgE in early childhood and ADHD. However, total IgE does not have an absolute association with any form of overt allergic/asthmatic disease, so to some extent, this might be expected (37). Furthermore, the prenatal period may be the period where the developing brain is more vulnerable to the maternal immune environment whereas IgE levels in childhood are more representative of other disease processes or biologic responses and exposures.

While this study does not specifically examine the mechanisms by which IgE (or the immune functions marked by IgE levels) may influence ADHD risk, we hypothesized that fetal neurodevelopment is influenced by maternal prenatal immune function. The concept of a link between the pattern of maternal prenatal immune function and ASD or ADHD has been discussed (14, 32, 38), yet as mentioned previously, studies have not specifically examined the role of maternal prenatal IgE in the development of ASD or ADHD in the offspring. The placenta is the critical interface between the mother and infant and IgE is not transported across the placenta as well as IgG. However, IgE is present in the human placenta and some studies have suggested that it may cross the placenta by forming immune complexes with IgG (39–41). In one study including 86 placentas, IgE was detected in all 86 placentas and appeared to be bound to placental macrophages (40, 41). In that study, the authors used a semiquantitative scoring system and found that placentas from atopic mothers did not have higher numbers of IgE cells (40). The histologic scoring system is highly subjective; thus, measurement error remains a possibility. Interestingly, placental macrophages are immunoprotective (42) and high concentrations of placental macrophages may be protective against ASD (16). The association with other developmental disorders needs to be evaluated. It is possible that there are differences in the prenatal exposure to IgE due to differences in the concentration or distribution of the placental macrophage as they bind IgE (40, 43). Additional studies are needed to understand the importance of IgE sequestered in the placenta and whether it enters fetal circulation, alters placental function, or alters inflammatory and cytokine response with neurodevelopment.

Our study has numerous strengths including a birth cohort that is socioeconomically and racially diverse. In addition, the cohort has a large number of prenatal biologic measures and data related to atopic disease. Pregnant women were not recruited based on disease history. Although we examined interactions with child sex, given that ADHD affects a greater number of males, this will be an important factor to further evaluate in future studies. While measurement of prenatal exposures in children who later develop ADHD are uncommon, it is also important to note that this study only included two measures of maternal total IgE (prenatal and 1 month postpartum), thus, we are unable to assess the changes in the total IgE during pregnancy. One previous study suggested that maternal total IgE changes differently during pregnancy in mothers with and without sensitization and allergic symptoms, but that study was small (*N* = 56) (44). In the WHEALS cohort, we previously reported that the total IgE increased a small amount between the prenatal and 1-month postpartum levels, but the change may have been due to increases in the maternal blood volume during pregnancy (45). Additional studies are needed to understand whether maternal IgE changes during pregnancy and how it could influence offspring neurodevelopment. In addition, future studies should consider how the maternal prenatal use of medications used to treat asthma and other allergic conditions influence neurodevelopment.

In summary, we found that maternal prenatal total IgE, but not early life or childhood levels of IgE, is associated with an increased risk of ADHD. This finding suggests that the prenatal period is an important window of exposure for risk of ADHD and, as such, might be a critical time period in which interventions to prevent these outcomes may be successful.

## Data Availability Statement

The raw data supporting the conclusions of this article will be made available by the authors, without undue reservation.

## Ethics Statement

All participants provided written, informed consent and study protocols were approved by the Henry Ford Health System Institutional Review Board; at the age 10–12 year visit, children provided written informed assent.

## Author Contributions

JS conceptualized and designed the study and drafted the initial manuscript. AS conducted the statistical analysis and drafted the initial manuscript. All authors contributed in revising and editing the manuscript, and approved the final version.

## Conflict of Interest

The authors declare that the research was conducted in the absence of any commercial or financial relationships that could be construed as a potential conflict of interest.

## Supplementary Material

Supplementary Figure 1 Distribution of untransformed and transformed IgE measurements.

Supplementary Table 1 Maternal and child characteristics associated with maternal total IgE levels.

## Abbreviations

Ig: immunoglobulin
Pre: prenatal
M: month
Y: Year.
