# Supplementary figures and images for: Prenatal IgE as a Risk Factor for the Development of Childhood Neurodevelopmental Disorders
Source: Front Pediatr. 2021 May 14;9:601092. doi: 10.3389/fped.2021.601092 (PMC8160239; doi:10.3389/fped.2021.601092)

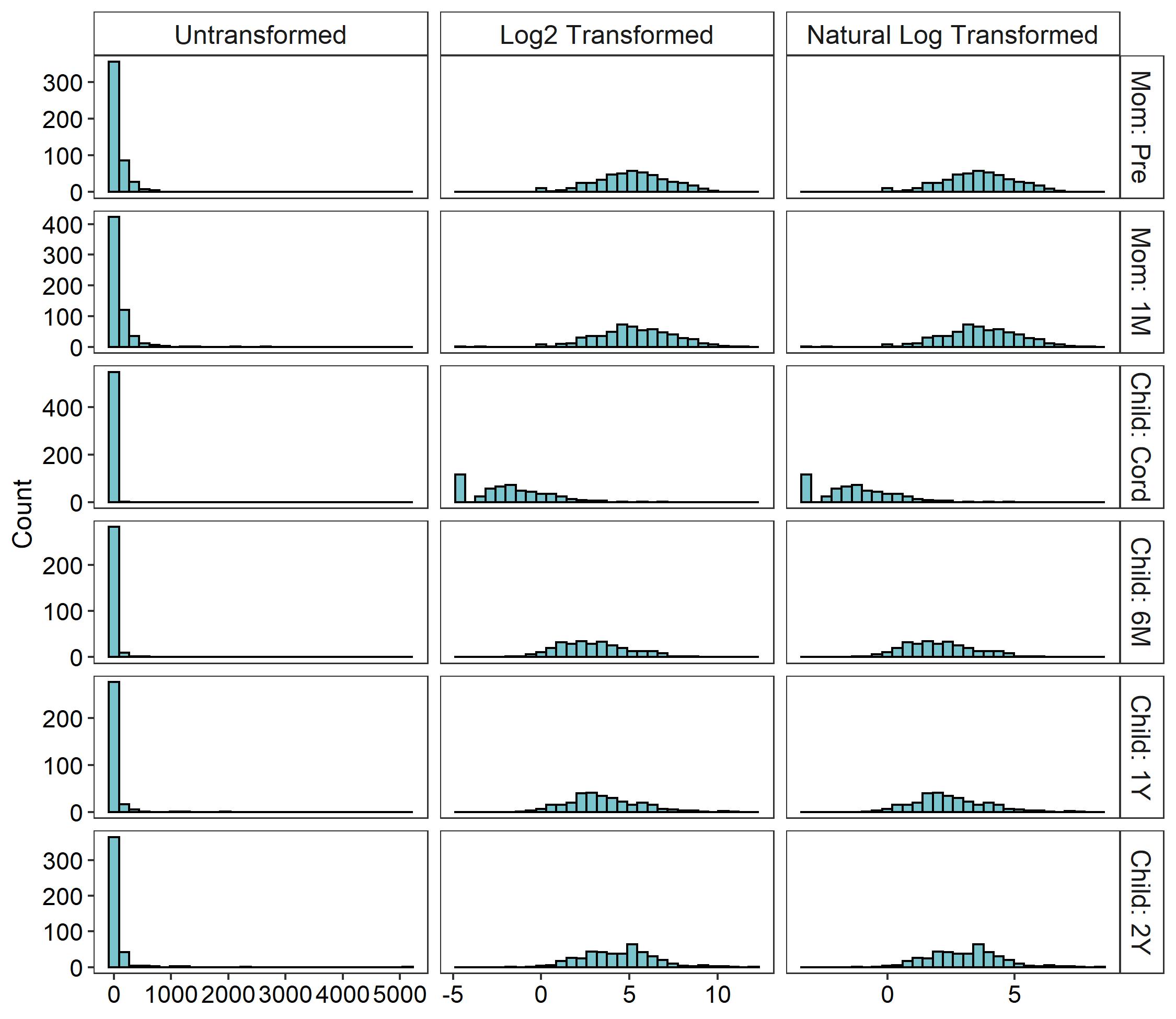

Supplement: Supplementary Figure 1 — Distribution of untransformed and transformed IgE measurements. [file Image_1.JPEG]
