# Supplementary material for: Prenatal IgE as a Risk Factor for the Development of Childhood Neurodevelopmental Disorders
Source: Front Pediatr. 2021 May 14;9:601092. doi: 10.3389/fped.2021.601092 (PMC8160239; doi:10.3389/fped.2021.601092)
Supplement: Supplementary Table 1 — Maternal and child characteristics associated with maternal total IgE levels. [file Table_1.DOCX]

**Supplementary Table 1. Maternal and child characteristics associated with maternal total IgE levels.**

| **Variable** | **Level** | **Maternal Total IgE: Prenatal^a^** | | | **Maternal Total IgE: 1-Month^a^** | | |
| --- | --- | --- | --- | --- | --- | --- | --- |
|  |  | **N** | **Mean or Pearson ρ** | **p-value^b^** | **N** | **Mean or Pearson ρ** | **p-value^b^** |
| Household income | <$20,000 | 46 | 3.37 | 0.33 | 61 | 3.38 | 0.20 |
|  | $20,000-<$40,000 | 108 | 3.72 |  | 140 | 3.79 |  |
|  | $40,000-<$80,000 | 138 | 3.52 |  | 164 | 3.50 |  |
|  | $80,000-<$100,000 | 61 | 3.37 |  | 85 | 3.73 |  |
|  | >$100,000 | 69 | 3.72 |  | 84 | 3.76 |  |
|  | Refused to answer | 62 | 3.81 |  | 71 | 3.91 |  |
| Mother married | No | 166 | 3.82 | **0.010** | 206 | 3.83 | 0.067 |
|  | Yes | 318 | 3.48 |  | 399 | 3.59 |  |
| Maternal education | <HS diploma | 17 | 3.38 | 0.052 | 22 | 3.76 | 0.19 |
|  | HS diploma | 67 | 3.57 |  | 90 | 3.56 |  |
|  | Some college | 229 | 3.77 |  | 276 | 3.81 |  |
|  | >Bachelor’s degree | 171 | 3.39 |  | 217 | 3.53 |  |
| Maternal age at birth (years) | --- | 484 | -0.095 | **0.038** | 605 | -0.024 | 0.55 |
| Maternal BMI-first measured in pregnancy | --- | 449 | 0.073 | 0.12 | 568 | 0.072 | 0.086 |
| Mom smoked during pregnancy | No | 447 | 3.56 | 0.10 | 553 | 3.65 | 0.33 |
|  | Yes | 37 | 3.97 |  | 52 | 3.87 |  |
| Prenatal ETS exposure | No | 374 | 3.56 | 0.34 | 460 | 3.64 | 0.41 |
|  | Yes | 110 | 3.71 |  | 145 | 3.76 |  |
| Prenatal indoor pets | No | 299 | 3.66 | 0.17 | 379 | 3.71 | 0.45 |
|  | Yes | 185 | 3.48 |  | 226 | 3.61 |  |
| Prenatal antibiotic use | No | 192 | 3.47 | 0.19 | 241 | 3.54 | 0.14 |
|  | Yes | 212 | 3.67 |  | 279 | 3.74 |  |
| Prenatal antifungal use | No | 326 | 3.51 | 0.057 | 422 | 3.57 | **0.021** |
|  | Yes | 78 | 3.86 |  | 98 | 3.97 |  |
| Child sex | Male | 242 | 3.73 | **0.033** | 302 | 3.82 | **0.020** |
|  | Female | 242 | 3.46 |  | 303 | 3.53 |  |
| Race-ethnicity of child | White | 105 | 3.17 | **<0.001** | 133 | 3.23 | **<0.001** |
|  | African American | 289 | 3.81 |  | 370 | 3.88 |  |
|  | Other/Mixed | 90 | 3.41 |  | 102 | 3.49 |  |
| First born child | No | 293 | 3.50 | 0.084 | 363 | 3.63 | 0.42 |
|  | Yes | 191 | 3.73 |  | 242 | 3.73 |  |
| Mode of delivery | Vaginal | 302 | 3.66 | 0.24 | 383 | 3.69 | 0.65 |
|  | C-Section | 181 | 3.50 |  | 221 | 3.64 |  |
| Gestational age at delivery (weeks) | --- | 480 | -0.034 | 0.46 | 598 | -0.061 | 0.14 |
| Birthweight z-score | --- | 459 | -0.093 | **0.046** | 573 | -0.068 | 0.10 |
| Breastfeeding status at 1-month | Not breastfed | 79 | 3.33 | 0.21 | 123 | 3.55 | 0.60 |
|  | Mixed feeding | 319 | 3.62 |  | 388 | 3.71 |  |
|  | Breastfeeding only | 66 | 3.72 |  | 86 | 3.62 |  |

BMI, body mass index; ETS, environmental smoke; HS, high school.

^a^Natural log transformed

^b^Analysis of variance p-value for categorical covariates and Pearson correlation p-value for numerical covariates.
